# Influence of Sociodemographic Factors on Stunting, Wasting, and Underweight Among Children Under Two Years of Age Born During the COVID-19 Pandemic in Central India: A Cross-Sectional Study

**DOI:** 10.7759/cureus.56381

**Published:** 2024-03-18

**Authors:** Naina Wakode, Kushagra Bajpai, Tanwi Trushna, Santosh Wakode, Kushagra Garg, Ankur Wakode

**Affiliations:** 1 Anatomy, Atal Bihari Vajpayee Government Medical College, Vidisha, IND; 2 Physiology, All India Institute of Medical Sciences, Bhopal, IND; 3 Physiology, All India Institute of Medical Sciences, Nagpur, IND

**Keywords:** covid-19 related issues, wasting, underweight, stunting, india, children

## Abstract

Introduction

The adverse effects of the coronavirus disease 2019 (COVID-19) pandemic on maternal and reproductive health extend beyond the immediate morbidity and mortality attributed directly to the disease. Pandemic-induced disruptions in the healthcare, social and economic infrastructures can exacerbate the already high burden of childhood undernutrition in India.

Method

A cross-sectional study was conducted in a selected district of the central Indian province of Madhya Pradesh. Data was collected from eligible children born during the COVID-19 pandemic (February 2020 to December 2021) who visited a selected tertiary care hospital for routine immunization during the study period. Weight-for-length, weight-for-age, and length-for-age were compared with reference values to obtain corresponding z-scores. Children with z-scores two standard deviations below the reference values were considered wasting, underweight and stunting, respectively. Descriptive statistics were employed to summarise the sociodemographic characteristics of participants. The association of sociodemographic, nutritional, and pregnancy-related factors with the z-scores were assessed via unpaired t-test and ANOVA.

Result

The studied 147 children were in the age group of nine to 29 months, of which 61 (58.1%) were males. Forty-two (28.6%) were found to be underweight, 22 (14.9%) had wasting and 51 (34.7%) were stunted. These prevalences were comparable to the estimates of the National Family Health Survey 2019-2021 (NFHS-5) for Madhya Pradesh and lower than the NFHS-4 (2015-2016), showing no discernible effect of being born during the pandemic on growth indicators. However, mothers' employment and family income were independent predictors of stunting whereas gestational age at birth, maternal education, and prolonged breastfeeding were all substantially linked with wasting in this study.

Conclusion

This study adds to the evidence base by reporting the prevalence of stunting, wasting and underweight along with their determinants in central India among children born during the COVID-19 pandemic. Our data did not reflect the expected increase in child malnutrition due to the COVID-19 pandemic-related disruptions in healthcare, social and economic infrastructure. Future research should incorporate the lessons learnt from our study to design a population-based study of under-five children and compare the prevalence of undernutrition in pandemic-born versus non-pandemic-born children.

## Introduction

The global pandemic caused by the severe acute respiratory syndrome coronavirus 2 (SARS-CoV-2) virus has had a profound impact on the global health system and socioeconomic infrastructure [1]. Particularly it has been acknowledged that the pandemic would have long-lasting negative effects on women around the globe [2]. Pregnancy is a period of marked physiological changes which make pregnant women and their foetuses especially vulnerable to the effects of the viral infection [3,4]. However, the adverse effects of the coronavirus disease 2019 (COVID-19) pandemic on maternal and child health are not limited to morbidity and mortality directly caused by the disease itself [5]. On one hand, the nationwide shutdowns had severe economic repercussions by disrupting livelihood activities and in turn worsening the socioeconomic status and nutrition of the poor [5]. On the other hand, health system disruptions and fear of seeking health care may also have affected the well-being of pregnant women and their babies [2,6,7]. In addition, social isolation, fear of contagion and the unknown and health safety concerns for their foetuses plagued mothers pregnant during the different waves of the COVID-19 pandemic leading to maternal psychological distress [8-10]. The cumulative impact of these multiple factors could culminate in adverse effects on the growing foetus [5,6,8] leading to adverse birth outcomes such as intrauterine growth restriction and low birth weight [11,12].

Children born during the COVID-19 pandemic, in addition to the aforementioned intrauterine insults, were also at higher risk of malnutrition postnatally [5]. Osendarp et al., 2021, predicted that pandemic-associated disruptions would precipitate wasting (defined as weight-for-height below two standard deviations (SD), reflecting acute malnutrition) and stunting (height-for-age below two SD due to chronic malnutrition) in an additional 9.3 million and 2.6 million children, respectively, residing in low- and middle-income countries (LMICs), including India [13]. Such adverse effects on early postnatal growth, particularly in the first two years of life, can have catastrophic consequences on the children predisposing them to overall higher mortality [14] and also on their communities by exacerbating loss of economic productivity and necessitating additional resource investment to mitigate these negative consequences [13].

Nevertheless, few studies have generated evidence regarding the adverse effects of being born during the pandemic on child growth. Shuffrey et al. in 2022 reported that birth during the pandemic was associated with delayed development, independent of maternal SARS-CoV-2 infection status, among American children [15]. Similar findings have been reported for Canadian and Irish infants [16,17]. There is an overall dearth of global data regarding growth indicators (wasting, stunting and underweight) among pandemic-born children which can independently affect childhood development. This knowledge gap is especially pronounced in a country like India which is already grappling with widespread child malnutrition even before the advent of the COVID-19 pandemic [18]. Keeping this in mind, this study focused on exploring the possible associations between socio-demographic factors and nutritional parameters with the growth status of pandemic-born children. The primary objectives of this study were to evaluate the proportion of children born during the COVID-19 pandemic in one district of the central Indian province of Madhya Pradesh who were wasted, stunted, and underweight as well as to identify the sociodemographic factors associated with their nutritional status.

## Materials and methods

Study setting

A cross-sectional study was conducted in the largest tertiary care hospital catering to the health needs of one district (Vidisha) of Madhya Pradesh (MP) in the central part of India. Madhya Pradesh is one of the eight low-performing provinces in India which have been categorized as Empowered Action Group (EAG) provinces [19,20]. As per the National Family Health Survey 2019-2021 (NFHS-5), MP recorded a neonatal mortality rate (NMR) of 29.0, infant mortality rate (IMR) of 41.3, and under-five mortality rate (UFMR) of 49.2, much higher than the Indian averages of 24.9, 35.2 and 41.9, respectively. Similarly, the percentage of children born in MP who had stunting, wasting, and were underweight (i.e., those with weight-for-age less than two SD) was 32.77%, 19.59%, and 32.39%, respectively, higher than the Indian average of 32.55%, 19.18%, 30.84%, respectively [21]. Of the 50 districts in MP, Vidisha has poor maternal and child health indicators [22].

Participants

To fulfill our objective, we calculated sample size using the formula for proportion in the online freely available OpenEpi calculator factoring in finite population correction while fixing confidence limits at 5% and power at 80%. We used the prevalence of total stunting (29.5) reported by an Indonesian mixed-methods study of pandemic-born children aged 0-23 months [23]. Accordingly, our calculated sample size was 137, and inflating the sample to account for non-response (10%) provided us with a target of 150 children. Of this target, we could enroll 147 children born during the COVID-19 pandemic (February 2020 to December 2021) who visited the selected tertiary care hospital for routine immunization during the study period. We excluded children with a diagnosed history of serious medical conditions such as congenital anomalies, those with moderate/severe intellectual disability, and finally children with mothers/caregivers with speech impediments that could potentially interfere with data collection.

Data collection tools

After obtaining approval from the institutional ethical committee, data was collected from children’s mothers/primary caregivers after administering an informed written consent. We collected socio-demographic factors related to the household and parents such as age, gender, education, family income, parents' working status, and occupation. In addition, we also recorded the details of the child including age and gender at the time of the hospital visit. The information on nutritional parameters like breastfeeding duration, the onset of solid food/weaning, and the onset of cow’s milk feed was collected since these are known to affect childhood growth [24]. We also noted the obstetrics and delivery history (gestational age at delivery, place and type of delivery, COVID-19 status during pregnancy). 

Since we included children coming to the immunization clinic, we relied on mother's self-reporting of their COVID-19 status during pregnancy. We categorized all women who provided a history of being infected with COVID-19 once or more during their pregnancy as being "positive" and those with no such history as "negative". In India, as per the guidelines issued under the National Health Mission by the Government of India, pregnant women reporting to any hospital for antenatal care/delivery were offered testing for COVID-19 by real-time reverse transcription-polymerase chain reaction (RT-PCR) and in case of unavailability of RT-PCR testing modality, approved kit-based Rapid Antigen Testing (RAT) was being done [25]. Hence, we assume that the 63 women who reported being COVID-19 positive during pregnancy would have undergone either of these two tests. 

Finally, anthropometric parameters (weight and length) of each child were measured following standard protocol. Briefly, after the removal of shoes and additional clothes, the child's weight was measured to the closest 0.1 kg using a calibrated electronic balance with a measurement range of up to 25 kg. The length of children aged six to 24 months was measured in a reclined position using an infantometer and read to the closest 0.1 cm. Before use, instrument calibration was tested. For example, the weighing scale was verified daily for accuracy against a standard weight.

Data analysis

Data was tabulated in Microsoft Excel 2016. Analysis was done using an add-in in Excel as well as the open-access biostatistics calculator provided by OpenEpi. For anthropometric analysis variables such as age, gender, weight, and length were used. These measurements were used to calculate the z-scores of weight-for-length, weight-for-age, and length-for-age. These generated indices were compared to standard reference values of the World Health Organization (WHO) to obtain the corresponding z-scores [26]. Children whose z-scores of length-for-age, weight-for-length, and weight-for-age were less than two SD from the median value of the reference population were considered stunted, wasted, and underweight, respectively. Descriptive statistics were employed to summarise the demographic features of the respondents. Continuous variables were expressed as mean along with SD whereas categorical variables were expressed as percentages. z-score of weight-for-length, weight-for-age, and length-for-age were compared with the socio-demographic data, nutritional parameters, and COVID-19 status of the mother during pregnancy by using unpaired t-test and ANOVA test. We set the cut-off for statistical significance at 5%.

## Results

A total of 147 children were enrolled in this study. The study participants were in the age group of nine to 29 months and 61 (58.1%) were males. Among the studied children, 42 (28.6%) were found to be underweight, 22 (14.9%) had wasting and 51 (34.7%) were stunted. As opposed to 105 (71.4%) children who had their weight in the normal range, 27 (29%) of all male babies and 15 (27.8%) of all female babies were underweight. The weight for length z-score within two SD below the reference (i.e., no wasting) was found in 75 (80.6%) males and 50 (92.6%) females. The remaining 18 (19.4%) male babies and four (7.4%) female babies had wasting. Similarly, stunting was seen in 33 (35.5%) of all male babies and 18 (33.3%) of all female babies. Table 1 shows the percentage of children of both genders with and without wasting, stunting and underweight.

**Table 1:** Distribution of study participants according to z-scores obtained for different growth indicators In this table, the number (percentage - %) of male and female children enrolled in this study whose z-scores of different growth indicators categories (weight-for-length, weight-for-age, and length-for-age) were found to be two standard deviations (SD) below the reference values provided by the World Health Organization (WHO) have been shown.

Category	Z Score < -2SD (Males)	Z Score < -2SD (Females)	Z Score > -2SD (Males)	Z Score > -2SD (Females)
Weight-for-length (n= 147)	18 (19.4%)	4 (7.4%)	75 (80.6%)	50 (92.6%)
Weight-for-age (n= 147)	27 (29%)	15 (27.8%)	66 (71%)	39 (72.2%)
Length-for-age (n= 147)	33 (35.5%)	18 (33.3%)	60 (64.5%)	36 (66.7%)

Analysing the effect of different sociodemographic and nutritional parameters on childhood wasting showed that lower levels of maternal education were associated with a higher chance of child wasting. In addition, gestational age at birth (term versus pre-term delivery) and the duration of breastfeeding (six months versus one year versus none) were significant predictors of the z-score for weight-for-length. However, we found no significant association was noted in the case of any of the other parameters like gender, family income, place of delivery, onset of cow milk, onset of solid food, COVID-19 status, type of delivery and working status of the mother (Table 2).

**Table 2:** Effect of sociodemographic and nutritional factors on weight-for-length z-scores In this table, the categories for each characteristic that have been used for analysis have been listed along with the number (percentage - %) of children falling in each category. Then, the mean and the standard deviations (SD) of weight-for-length z-scores [compared to standard reference values of the World Health Organization (WHO)] for each category of children have been shown. The last column shows the p-values (rounded to two decimal places) obtained in the analysis comparing the z-scores with other characteristics by using the unpaired t-test and ANOVA test. Statistically significant associations have been shown in bold.

Characteristic	Category	Number (%)	Weight-for-length (Mean ± SD)	P value
Gender	Male	93 (63.2%)	-0.7 ± 1.41	0.12
	Female	54 (36.7%)	-0.6 ± 1.16	
Family Income	Less than 10000 Indian Rupees	107 (72.8%)	-0.6 ± 1.39	0.22
	Between 10000 to 50000 Indian Rupees	40 (27.2%)	-0.75 ± 1.17	
Delivery	At Home	5 (3.4%)	-0.3 ± 1.65	0.37
	In Hospital	142 (96.6%)	-0.7 ± 1.32	
Mother education	Graduate	37 (25.2%)	-0.7 ± 1.28	0.02
	School	95 (64.6%)	-0.6 ± 1.29	
	Illiterate	15 (10.2%)	-0.9 ± 1.96	
Gestational age at birth	Term	134 (91.1%)	-0.7 ± 1.28	0.05
	Preterm	13 (8.8%)	-0.9 ± 1.81	
Breastfeeding duration	More than six months	95 (64.6%)	-0.7 ± 1.29	0.03
	Continue for one year	44 (29.9%)	-0.7 ± 1.26	
	Not provided	8 (5.4%)	-0.85 ± 2.04	
Onset of Cow Milk	Before six months	13 (8.8%)	-0.7 ± 1.19	0.38
	After six months	76 (51.7%)	-0.8 ± 1.32	
	Not yet provided	58 (39.4%)	-0.55 ± 1.44	
Onset of Solid food	Before six months	71 (48.3%)	-0.7 ± 1.41	0.49
	After six months	66 (44.9%)	-0.7 ± 1.23	
	Not yet provided	10 (6.8%)	-0.6 ± 1.47	
Maternal COVID-19 status during pregnancy	Positive	63 (42.8%)	-0.5 ± 1.32	0.95
	Negative	84 (57.1%)	-0.8 ± 1.33	
Type of delivery	Normal Vaginal Delivery	102 (69.4%)	-0.7 ± 1.37	0.50
	Caesarean section	45 (30.6%)	-0.7 ± 1.25	
Maternal Occupation	Working	21 (14.3%)	0.2 ± 1.14	0.57
	Housewife	126 (85.7%)	-0.75 ± 1.28	

Our study estimated that children belonging to low-income families (family monthly income less than 10000 rupees) and those with working mothers were at higher risk of stunting. However, none of the other studied parameters had any significant effect on stunting (Table 3). Similarly, we found no significant association between weight-for-age z-score (indicative of underweight) and sociodemographic/nutritional factors like gender, education of mother, family income, working status of parents, gestational age at birth, duration of breastfeeding, onset of cow milk feed, COVID-19 status during pregnancy, type, and place of delivery (Table 4).

**Table 3:** Effect of sociodemographic and nutritional factors on length-for-age z scores In this table, the categories for each characteristic that have been used for analysis have been listed along with the number (percentage - %) of children falling in each category. Then, the mean and the standard deviations (SD) of length-for-age z-scores [compared to standard reference values of the World Health Organization (WHO)] for each category of children have been shown. The last column shows the p-values (rounded to two decimal places) obtained in the analysis comparing the z-scores with other characteristics by using the unpaired t-test and ANOVA test. Statistically significant associations have been shown in bold.

Characteristic	Category	Number (%)	Length-for-age (Mean ± SD)	P value
Gender	Male	93 (63.2%)	-1.6 ± 1.69	0.43
	Female	54 (36.7%)	-1.5 ± 1.54	
Family Income	Less than 10000 Indian Rupees	107 (72.8%)	-1.75 ± 1.35	0.05
	Between 10000 to 50000 Indian Rupees	40 (27.2%)	-1.35 ± 1.72	
Delivery	At Home	5 (3.4%)	-1.31 ± 2.5	0.10
	In Hospital	142 (96.6%)	-1.6 ± 1.61	
Mother education	Graduate	37 (25.2%)	-1.3 ± 1.36	0.25
	School	95 (64.6%)	-1.6 ± 1.72	
	Illiterate	15 (10.2%)	-1.8 ± 1.52	
Gestational age at birth	Term	134 (91.1%)	-1.55 ± 1.61	0.53
	Preterm	13 (8.8%)	-2 ± 1.78	
Breastfeeding duration	More than six months	95 (64.6%)	-1.4 ± 1.49	0.13
	Continue for one year	44 (29.9%)	-1.85 ± 1.93	
	Not provided	8 (5.4%)	-1.7 ± 1.65	
Onset of Cow Milk	Before six months	13 (8.8%)	-1.55 ± 1.51	0.47
	After six months	76 (51.7%)	-1.2 ± 1.56	
	Not yet provided	58 (39.4%)	-1.75 ± 1.77	
Onset of Solid food	Before six months	71 (48.3%)	-1.3 ± 1.43	0.22
	After six months	66 (44.9%)	-1.6 ± 1.76	
	Not yet provided	10 (6.8%)	-2.85 ± 1.47	
Maternal COVID-19 status during pregnancy	Positive	63 (42.8%)	-1.8 ± 1.75	0.22
	Negative	84 (57.1%)	-1.35 ± 1.52	
Type of delivery	Normal Vaginal Delivery	102 (69.4%)	-1.6 ± 1.64	0.95
	Caesarean section	45 (30.6%)	-1.5 ± 1.62	
Maternal Occupation	Working	21 (14.3%)	-2 ± 2.06	0.05
	Housewife	126 (85.7%)	-1.5 ± 1.55	

**Table 4:** Effect of sociodemographic and nutritional factors on weight-for-age z-scores In this table, the categories for each characteristic that have been used for analysis have been listed along with the number (percentage - %) of children falling in each category. Then, the mean and the standard deviations (SD) of weight-for-age z-scores [compared to standard reference values of the World Health Organization (WHO)] for each category of children have been shown. The last column shows the p-values (rounded to two decimal places) obtained in the analysis comparing the z-scores with other characteristics by using the unpaired t-test and ANOVA test.

Characteristic	Category	Number (%)	Weight-for-age (Mean ± SD)	P value
Gender	Male	93 (63.2%)	-1.3 ± 1.34	0.23
	Female	54 (36.7%)	-1.25 ± 1.15	
Family Income	Less than 10000 Indian Rupees	107 (72.8%)	-1.3 ± 1.31	0.36
	Between 10000 to 50000 Indian Rupees	40 (27.2%)	-1 ± 1.15	
Delivery	At Home	5 (3.4%)	-2.3 ± 1.91	0.12
	In Hospital	142 (96.6%)	-1.3 ± 1.25	
Mother education	Graduate	37 (25.2%)	-1 ± 1.21	0.9
	School	95 (64.6%)	-1.3 ± 1.29	
	Illiterate	15 (10.2%)	-1.7 ± 1.25	
Gestational age at birth	Term	134 (91.1%)	-1.15 ± 1.22	0.09
	Preterm	13 (8.8%)	-2.1 ± 1.67	
Breastfeeding duration	More than six months	95 (64.6%)	-1.2 ± 1.25	0.32
	Continue for one year	44 (29.9%)	-1.45 ± 1.22	
	Not provided	8 (5.4%)	-0.6 ± 1.80	
Onset of Cow Milk	Before six months	13 (8.8%)	-1.3 ± 1.29	0.7
	After six months	76 (51.7%)	-1.3 ± 1.36	
	Not yet provided	58 (39.4%)	-1.1 ± 1.21	
Onset of Solid food	Before six months	71 (48.3%)	-1 ± 1.28	0.95
	After six months	66 (44.9%)	-1.3 ± 1.24	
	Not yet provided	10 (6.8%)	-2.2 ± 1.32	
Maternal COVID-19 status during pregnancy	Positive	63 (42.8%)	-1.3 ± 1.31	0.64
	Negative	84 (57.1%)	-1.2 ± 1.24	
Type of delivery	Normal Vaginal Delivery	102 (69.4%)	-1.3 ± 1.25	0.60
	Caesarean section	45 (30.6%)	-1.3 ± 1.33	
Maternal Occupation	Working	21 (14.3%)	-0.8 ± 0.99	0.17
	Housewife	126 (85.7%)	-1.3 ± 1.29	

## Discussion

This study was conducted to assess the growth of children born during the COVID-19 pandemic. The early years of life are a critical period for assessing the onset of malnutrition or obesity and implementing management strategies [27]. Therefore, we focussed on pandemic-born children who were less than or equal to 24 months of age. The weight of children naturally varies during growth, therefore weight-for-length, weight-for-age, and length-for-age estimation were taken into consideration in our study and we reported z-scores of Indian children born during the COVID-19 pandemic compared with the WHO child growth standards [28].

Stunting, defined as length-for-age two standard deviations below the median values as per WHO child growth standards [29,30], is considered one of the best indicators of child health [31]. Globally, 149.2 million children under five are affected by stunting, with more than 50% of them residing in Southern Asia, including India [32]. In our study, we found that approximately 51 (34.7%) of the studied children were stunted. Our findings are in line with the NFHS-5 (2019-2021), which reports that 32.77% and 32.55% of children in MP and all over India have stunting, respectively [21]. Stunting is reported to be the result of chronic inadequate maternal and child nutrition that can occur secondary to multiple factors ranging from socioeconomic status influenced by the parent’s education/occupation to the child’s medical history affected by recurrent infections, hospitalizations, or chronic diseases that lead to poor intake, absorption, and utilization of foods [27]. Therefore, in our present study of pandemic-born children, children belonging to low-income families and those with working mothers were found to be at higher risk of stunting, which is in line with published evidence. For example, Prasad et al. in 2021 reported that children of households with low socioeconomic status had two times higher odds of stunting when compared to children born in rich households. The authors also highlighted the adverse effects of mothers’ working status [33]. Similar findings were reported by another study conducted in Bangladesh which showed that the odds of stunting were two times higher in children born to mothers actively engaged in occupations than those with non-working mothers [34]. Multiple reasons could be determining this association. Faced with the lack of alternate caregivers and inadequate family support, inadequacy of time due to additional occupational commitments could compel the mothers to ignore household/childcare chores and health seeking leading to poorer household hygiene, poor child feeding practices, incomplete vaccinations, and increased risk of infectious diseases among their children [34]. However, more research needs to be conducted to explore these issues and gain a better understanding of the mechanisms at play, particularly in the Indian context, to enable the designing and implementation of appropriate interventions.

Our findings show that 22 (14.9%) of the studied children had wasting while 42 (28.6%) were found to be underweight. Our results are lower when compared to the NFHS-5 reports of wasting and underweight for children born in MP (19.59% and 32.39%) and the Indian average of 19.18% and 30.84% [21]. Such differences at the sub-national level are expected particularly for wasting, which is an indicator of acute malnutrition owing to the multitude of differences across communities [30]. Therefore, stunting, or short length-for-age, is often considered one of the best indicators of child health [31].

Exploration of the reasons behind acute malnutrition manifesting as wasting, which could even be fatal for the child patient, showed a significant positive relationship between children who completely breastfed beyond six months and diagnosis of wasting. Although it is widely acknowledged that exclusive breastfeeding until six months of age is beneficial for child growth and development [35], the effect of prolonged breastfeeding is actively debated. While some researchers have reported no benefits of prolonged breastfeeding [36], others have reported adverse effects on child growth [37]. It has been explained that when children on prolonged breastfeeding might not get the requisite nourishment for their developing bodies and hence exhibit signs of wasting caused by acute dietary deprivation [38].

We also identified other factors affecting the occurrence of wasting in our study such as maternal education level and gestational age at birth. The chances of wasting were higher in children born to uneducated mothers than among children of college-educated mothers. This result is similar to the findings of previous research [33]. In our study, wasting was significantly linked to gestational age at birth of children which resonates with published evidence that has highlighted the effect of preterm birth and low birth weight on child growth and development [21,39]. Considering the heavy burden of childhood undernutrition in India, the identification of factors contributing to its persistence is important and the findings generated by our study would enrich the evidence base and provide direction for future intervention studies.

The need for ameliorating the burden of childhood malnutrition cannot be overstated because undernourished children, especially those with stunting, face the burden of potentially irreversible adverse consequences on physical and neurocognitive development and even increased risk of degenerative diseases in adulthood [27]. Therefore, under goal two of the sustainable development goals (SDGs), indicator 2.2.1 emphasizes the reduction in the prevalence of stunting in children under five by 2025 [29]. To fulfill this goal, multiple schemes and programs are being run by the central and provincial governments in India including the Infant and Young Child Feeding Guidelines implemented through the National Breastfeeding Promotion Program and the Integrated Child Development Scheme, the National Nutrition Mission (Poshan Abhiyaan) and the Mid-day Meal Scheme for school-going children, to name a few [18], in addition to the routine health system services targeting maternal and child health.

Disruptions caused by the COVID-19 pandemic in these existing systems are expected to have exacerbated child malnourishment in India [13]. This is especially true for stunting owing to its multifactorial etiology [27]. However, the proportion of stunted children found in our study is lower than the data reported for population-level stunting (i.e., 41.1) in the Vidisha district in the previous round of NFHS-4 (2015-16) [40]. There could have been many reasons behind this observed decreasing trend in stunting instead of the anticipated increase in malnutrition in the pandemic birth cohort. For example, Dhillon et al. in 2023 confirmed that even during the pandemic community health workers (CHWs) like accredited social health activists (ASHA), auxiliary nurse midwives (ANM), and Anganwadi workers (AWW) were actively delivering antenatal care, nutritional services, and health promotion-related counseling to pregnant women in India [41] and thus, the expected COVID-19-induced disruption of these essential services was successfully averted. In addition, exposure to air pollutants is widely acknowledged as one of the important risk factors for wasting and stunting in children under five [42-44]. Even Indian evidence for the same is gradually accruing [45-47]. During COVID-19 lockdowns, urban ambient air quality significantly improved [48] and thus, the burden of childhood malnutrition attributable to air pollution would have decreased. This reduction could have counteracted and masked any pandemic-related increase in undernutrition. Therefore, we recommend future research to account for these factors.

The results of this study ought to be interpreted while keeping in mind its limitations. We were unable to collect information from an appropriate control group of children born after the COVID-19 pandemic was officially declared to have ended. Such information while accounting for changes in other factors as mentioned previously would have enabled us to generate conclusive quantitative evidence about the exact change in child wasting, stunting, and underweight prevalence during the pandemic. Secondly, we only used anthropometry to diagnose undernutrition. The use of body composition, such as lean and fat tissue, can provide additional information on the physiological and functional relationship between wasting and stunting. Further, our study was conducted in a tertiary care hospital and thus, the patients enrolled in this study, by virtue of their easy access to healthcare, might not be representative of the community. It is therefore feasible that the actual burden of child malnutrition might be higher in the community. It would be worthwhile to conduct a future population-based study of a larger sample of children under five and compare the prevalence of undernutrition in pandemic-born versus non-pandemic-born children. Finally, future studies would also benefit from the inclusion of in-depth analytic techniques such as regression analysis to account for the effect of confounders and covariates on the dependent variables.

## Conclusions

This study adds to the evidence base by reporting the prevalence of stunting, wasting and underweight in central India among children born during the COVID-19 pandemic. Our findings contribute to the theoretical understanding of the determinants of stunting and wasting rooted in socioeconomic and nutritional perspectives. We have also provided evidence regarding the adverse effects of prolonged breastfeeding on childhood growth to help in the ongoing debate. We have reiterated the negative consequences on child nutrition of low maternal education and the involvement of mothers in active occupation without adequate family support. Finally, our data did not reflect the expected increase in child malnutrition due to the COVID-19 pandemic-related disruptions in healthcare, social and economic infrastructure. However, our findings could not differentiate between the negative effects of the pandemic from the unintended decrease in air pollution and the consequent improvement in child health indicators. Future research should incorporate the lessons learnt from our study to design a population-based study of children under five and compare the prevalence of undernutrition in pandemic-born versus non-pandemic-born children.
